# Evaluation of different scoring systems and gene mutations for the prognosis of myelodysplastic syndrome (MDS) in Chinese population

**DOI:** 10.7150/jca.30363

**Published:** 2020-01-01

**Authors:** Meng-yi Du, Min Xu, Jun Deng, Lin Liu, Tao Guo, Ling-hui Xia, Yu Hu, Heng Mei

**Affiliations:** 1 Institute of Hematology, Union Hospital, Tongji Medical College, Huazhong University of Science and Technology, Wuhan 430022, China.; 2 Collaborative Innovation Center of Hematology, Huazhong University of Science and Technology, Wuhan, Hubei 430022, PR China.

**Keywords:** myelodysplastic syndrome, risk assessment, prognosis, gene mutations

## Abstract

MDS is a heterogeneous disease with diverse clinical manifestations, and an effective prognostic evaluation tool for MDS patients is needed. To achieve more accurate prognosis assessment for Chinese MDS patients, here we examined several scoring systems and explored the implications of gene mutations. The prognostic conditions were stratified against three different score systems (International Prognostic Scoring System (IPSS), WHO Prognostic Scoring System (WPSS), and Revised International Prognostic Scoring System (IPSS-R)) were retrospectively applied to 110 de novo MDS patients in study cohort in our hospital and the prognostic conditions were stratified respectively. IPSS-R out-performed the others, since it had less overlaps in survival curve, especially in the relatively low-risk group. Furthermore, genetic mutations were identified in 84 out of 110 patients and their association with overall survival (OS) were determined. Among them, sixty-three percent patients had at least one-point mutation, including thirty-five patients with normal karyotypes. The presence of TP53 mutations, but not TET2, DNMT3A or ASXL1 mutations was significantly correlated with shorter OS. A new model incorporating IPSS-R and TP53 mutations into survival analysis was proposed, and the prognostic value of this model was validated to be predominant in a 190-primary MDS patient independent cohort. Our data suggested that IPSS-R was more suitable for Chinese population. Attentions should be paid to the unfavourable mutations that might exert impact on the survival, especially in patients with relatively low risk.

## Introduction

The myelodysplastic syndrome (MDS) represents a heterogeneous group of clonal hematopoietic disorders with diverse clinical manifestations. Approximately, 20%-30% of MDS patients may progress to acute myeloid leukemia 1, 2. Clinically, accurate prediction of its prognosis is the cornerstone of guiding optimal MDS treatment. Prognostic models are useful tools for assessing risk of leukemic transformation, predicting life expectancy and making treatment plans 3. Over the past two decades, three different scoring systems (IPSS^4^, WPSS^5^ and IPSS-R 6-8) have been developed and successfully used worldwide in clinical practice for the evaluation of MDS prognosis^9^. Nonetheless, each system has its limitations and great divergences exists in various institutions when choosing a system for application 10. All three systems cannot accurately assess cytogenetically normal patients at relatively low-risk^8^, ^11^. Moreover, MDS prognosis varies greatly with genetic profiles and races^12^-^15^. In this study, we aimed to identify the best scoring system for Chinese MDS patients by comparing the abovementioned ones. Importantly, we determined genetic mutations in cytogenetically normal patients to provide more accurate prognostic stratification.

## Materials and methods

### General information

A total of 300 patients, 110 for study cohort and 190 for validation cohort, with primary MDS were enrolled from the Institute of Hematology, Union Hospital, Tongji Medical College, Huazhong University of Science and Technology, Wuhan, China. Patients diagnosed with MDS were retrospectively collected from Jan. 2014 to Dec. 2018. This study has been approved by the Ethics Committees of Union Hospital, Tongji Medical College, Huazhong University of Science and Technology, Wuhan, China. Written informed consents were obtained from the patients or, in some special cases, from their relatives, and the study was conducted in strict accordance to the Helsinki Declaration (2013). The follow-up started from the date of enrolment to Jun.2017 for study cohort or Jun. 2019 for validation cohort or death and was conducted by telephoning patients or their attending doctors. In our series, no case was lost in the follow-up study. Patient characteristics were collected, including age, sex, initial peripheral blood data (absolute neutrophil count (ANC), hemoglobin (HB), platelet count (PLT)), initial percentage of bone marrow (BM) blasts, karyotype, WHO classification and survival status. Data concerning treatments received by patients during the follow-up period were recorded.

### Diagnostic criteria

MDS diagnosis was made if (1) stable cytopenia ≥ 6 months (if stable cytopenias lasted for only 2 months, a specific karyotype or bi-lineage dysplasia were needed); (2) other potential disorders that are likely to trigger dysplasia and/or cytopenia were ruled out; (3) equal or greater than one of the three following requirements were satisfied: ① dysplasia (one or more of the 3 major bone marrow lineages≥10%); ② blast cell count ranging between 5% to 19%; ③ a specific MDS-associated karyotype [e.g., del(5q), del(20q), +8, or -7/del(7q)].

Furthermore, we used several other indicators that might help diagnose MDS, including aberrant immune-phenotypes, bone marrow abnormalities, or the presence of some molecular markers, signs or manifestations (i.e. abnormal CD34 antigen expression, fibrosis, dysplastic megakaryocytes, atypical localization of immature progenitors, and myeloid clonality)^16^.

### Methods

Three scoring systems (IPSS/WPSS/IPSS-R) were assessed in 110 study-cohort patients accordingly, in terms of mortality and median survival time. Then, the next genetic sequencing (NGS) tests were done in 84 out of 110 patients. Incidence of gene mutations and pattern of co-existing mutations were calculated, the associations of mutations with phenotypes and OS were determined. Moreover, the validation of a new prognostic model established by professor HOU and his colleges 17(HOU model) was taken, and an integrated scoring system based on our data comprehending IPSS-R and TP53 mutations (DU model) into survival analysis was proposed by us. The prognostic value of IPSS-R, HOU model and our model was then tested in the validation cohort of 190 primary MDS patients. Blood test, bone marrow cell morphology, bone marrow biopsy, cytogenetics and other tests were conducted in the patients. Patients in all groups received the same examinations and tests.

### Grouping

According to the WHO (2008) classification 18, patients in our serious were classified into 5 categories in terms of myelogram and blood picture (Table 1). We didn't divide patients into groups according to WHO (2016) classification^19^, because most of them were diagnosed before the year of 2016. Additionally, patients were grouped according to IPSS, WPSS and IPSS-R, by following the guidance of the 2017.V2 NCCN (Table 2). Relatively low-risk group referred to low-risk group and intermediate-1 group against IPSS; very low-risk group, low-risk group and intermediate risk group against WPSS; very low-risk group, low-risk group and intermediate risk group (IPSS-R intermediate patients may be managed as lower risk if their score is ≤3.5) against IPSS-R. The other subgroups against individual systems were designated as relatively high-risk group. All data used to calculate the risk stratification of the prognosis was the data at the time of diagnosis.

### Gene sequencing

2-3 ml bone marrow was collected from 84 of 110 study-cohort and 190 validation-cohort MDS patients and then heparinized. Mononuclear cells were harvested from this bone marrow samples by using Ficoll solution. Then DNA was extracted from mononuclear cells using DNA extraction kit (Tiangen Biochemical Technology Co. Ltd, Beijing, China), and preserved at 2-8°C.

A total of 25 well-known genes from the MDS genomes (Figure 2.) were analysed by targeted deep sequencing. Genomic DNA from each sample was sequenced. Briefly, KAPA hyper library kit and corresponding adaptors were used to construct library, and then Agencourt AMPure XP reagent (Beckman Coulter, Inc.) was used to purify magnetic beads. The sample was mixed with MiSeq Reagent Kit V3 (600 cycles)/MiSeq Reagent Kit V2 (500 cycles) or FC-420-1004 MiniSeq Mid Output Kit (300 cycles)/Miniseq High Output Kit (300 cycles) (Yuanqi Biopharmaceutical Technology Co. Ltd., Shanghai, China), and then sequenced on an illumina MiSeq or illumina MiniSeq sequencer (Illumina, California, American), respectively. Read pairs were aligned to Refseq hg19 (downloaded from UCSC Genome Browser) by Burrows-Wheeler Aligner (BWA) version 0.7.13-r1126. Samtools version 1.3 was used to generate chromosomal coordinate-sorted bam files. The mean depth of each sample was 1500x-2000x, with an average >90% of the target sequence which covered sufficiently deep for variant calling. The designed sequencing primers covered all mutational hot spots of the candidate genes. Samtools mpileup were applied to call single nucleotide variants (SNVs) and indels. Homemade pipeline was used to filter SNVs and indels detected by the above software, excluding: 1) mutations reported with low confidence; 2) mutations reported in 1000 Genomes (database of single nucleotide polymorphism (dbSNP) 138) as common single nucleotide polymorphisms (SNPs) and not included in COSMIC version v83. SNVs and indels were annotated using Annovar Software. All the somatic functional mutations, including nonsynonymous SNVs, frameshift or in-frame indels, stop-gain and stop-loss were obtained. Visual inspection was used to exclude potential false positive results. Finally, Integrative Genomics Viewer (http://www.igv.org/) software was used for data visualization and verification.

### Treatments

The treatment for relatively low-risk group of patients primarily aimed to optimize their hematopoietic function and improve the quality of life. Patients with HB <60 g / L or severe anemia were infused with suspended erythrocytes. Patients with PLT <20*10^9 / L or suffering from active bleeding were given hemostatic drugs or transfused with platelets. Patients with isolated del (5q-) were administered thalidomide. Severe agranulocytosis was treated with colony-stimulating factor and iron, among others. The treatment for high-risk MDS was aimed to delay disease progression and prolong survival. Patients above 65 or in poor conditions were given demethylating drugs (decitabine). Patients under 65 in relatively stable conditions were treated with decitabine in combination with CAG regimen (aclarubicin, cytosine arabinoside, and granulocyte colony stimulating factor).

### Statistical analysis

The comparison of median survival time was performed using the Tarone-Ware test. Survival analysis started from the date of diagnosis to the date of death or last contact. Survival curves were prepared by employing the Kaplan-Meier method. Spearman correlation analysis was used to ascertain how phenotypes correlated with mutations. Proportions of mutational co-existence were compared by utilizing Chi-square test. Cox proportional hazard regression model was used for univariable and multivariable analysis. A P<0.05 was considered to be statistically significant. SPSS 22.0 software package (version 22; IBM Corporation, Armonk NY) was used for statistical analysis.

## Results

### Follow-up results of study-cohort

The series included 60 males and 50 females, with a male-to-female ratio at 1.2:1. The median age of these patients was 49.5 years old (Standard Deviation (SD) =15.8), ranging from 13 to 89. All the patients were followed up for an average of 11 (4 ~ 30) months. Forty-three patients died, with a mortality of 39.0%. Eight cases converted into sAML, with the conversion rate at 7.3% (Table 1). Furthermore, we evaluated the outcome of different prognostic scorning system. We found that risk stratification using IPSS-R showed the best prognostic values as the worse the prognosis the higher the mortality in patients. In contrast, IPSS and WPSS were somehow deficient in predicting prognosis because high mortality was observed in the lowest risk group using these two systems respectively (Table 2).

### Survival analysis

**Figure 1:**
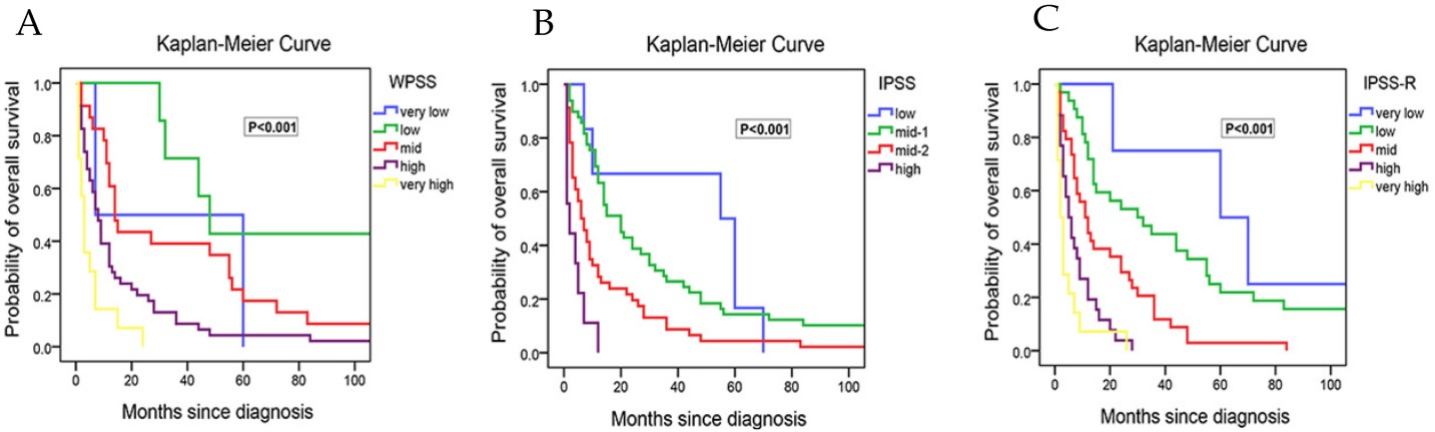
Kaplan-Meier curves for overall survival of 110 study cohort patients in our study with survival data. (A) Survival of patients stratify according to WPSS. (B) Survival of patients stratify according to IPSS. (C) Survival of patients stratify according to IPSS-R.

We next performed the survival analysis. We found that the median survival time was reduced progressively with the increased risk stratified according to IPSS, WPSS and IPSS-R, respectively (Table 2). The differences in the median survival time among strata within the system were of statistical significance. However, the median OS of the relatively low risk groups in all three scoring systems were significantly shorter than the referenced data in NCCN Guideline^1^. For example, the median OS of low-risk group in IPSS-R was 63 months in NCCN Guideline and 35 months in our study. Moreover, the survival curve showed that the differences in prognosis among relatively low-risk groups according to WPSS were not evident (Figure 1A). The possible explanation is that there were many intersections between relatively low risk groups, only the very-high risk group did not cross with others, making it difficult to differentiate the prognosis in the low-risk groups. As for IPSS, there were fewer intersections among subgroups, but crossover still existed between low risk group and intermediate-1 risk group (Figure 1B). Besides, early death existed in the lowest risk group of both IPSS and WPSS. In contrast, IPSS-R had least intersections with clear survival curves for each group, which rendered it easy to judge the prognostic differences among different risk groups (Figure 1C).

### Incidence of gene mutations and pattern of co-existing mutations

**Figure 2:**
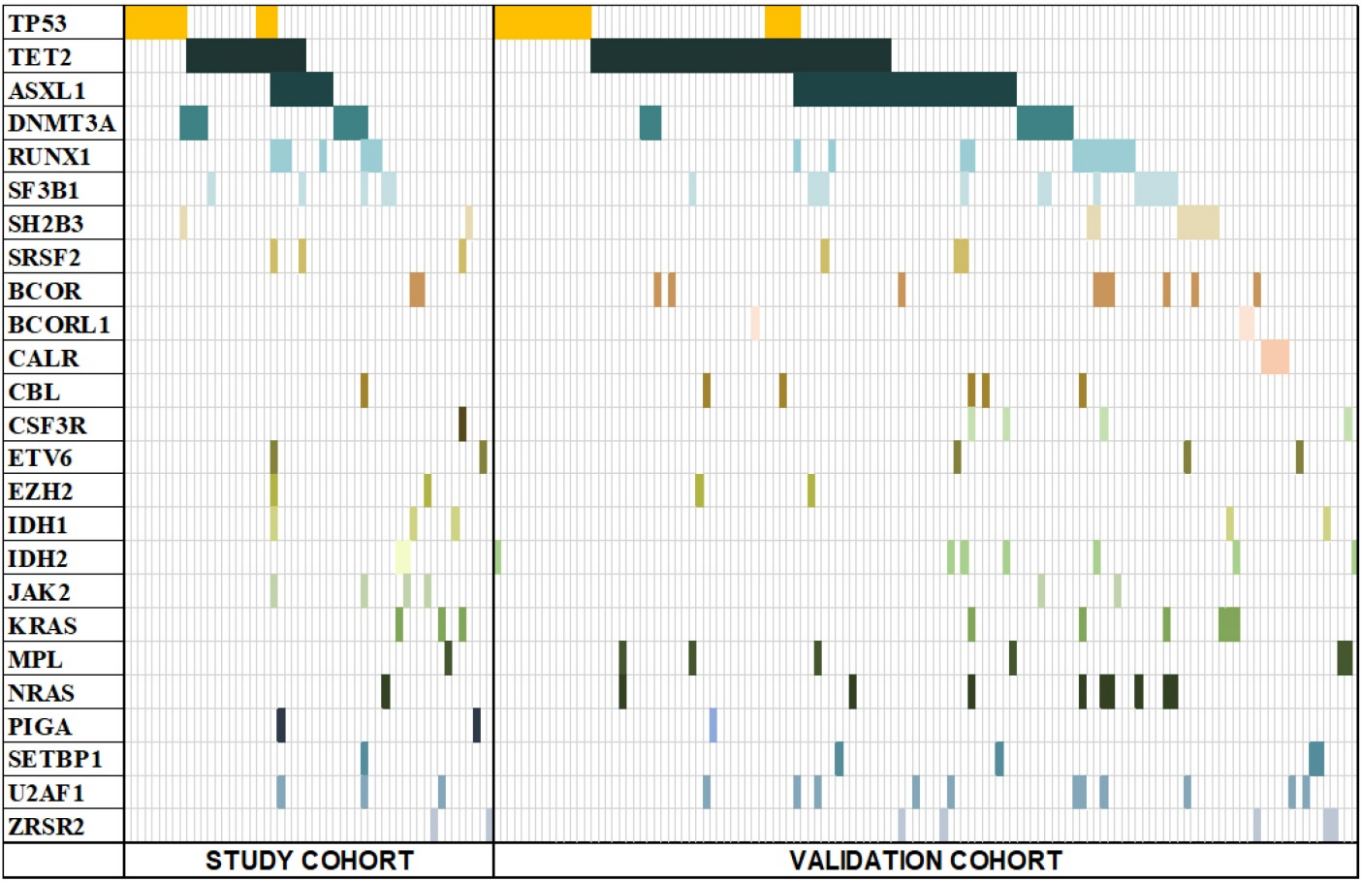
Mutation spectrum of both study cohort and validation cohort. 53 out of 84 study-cohort and 124 out of 190 validation-cohort patients had at least one mutation of the 25 MDS-associated genes. Each column represents an individual patient sample, and each coloured cell represents a mutation of the gene.

**Figure 3:**
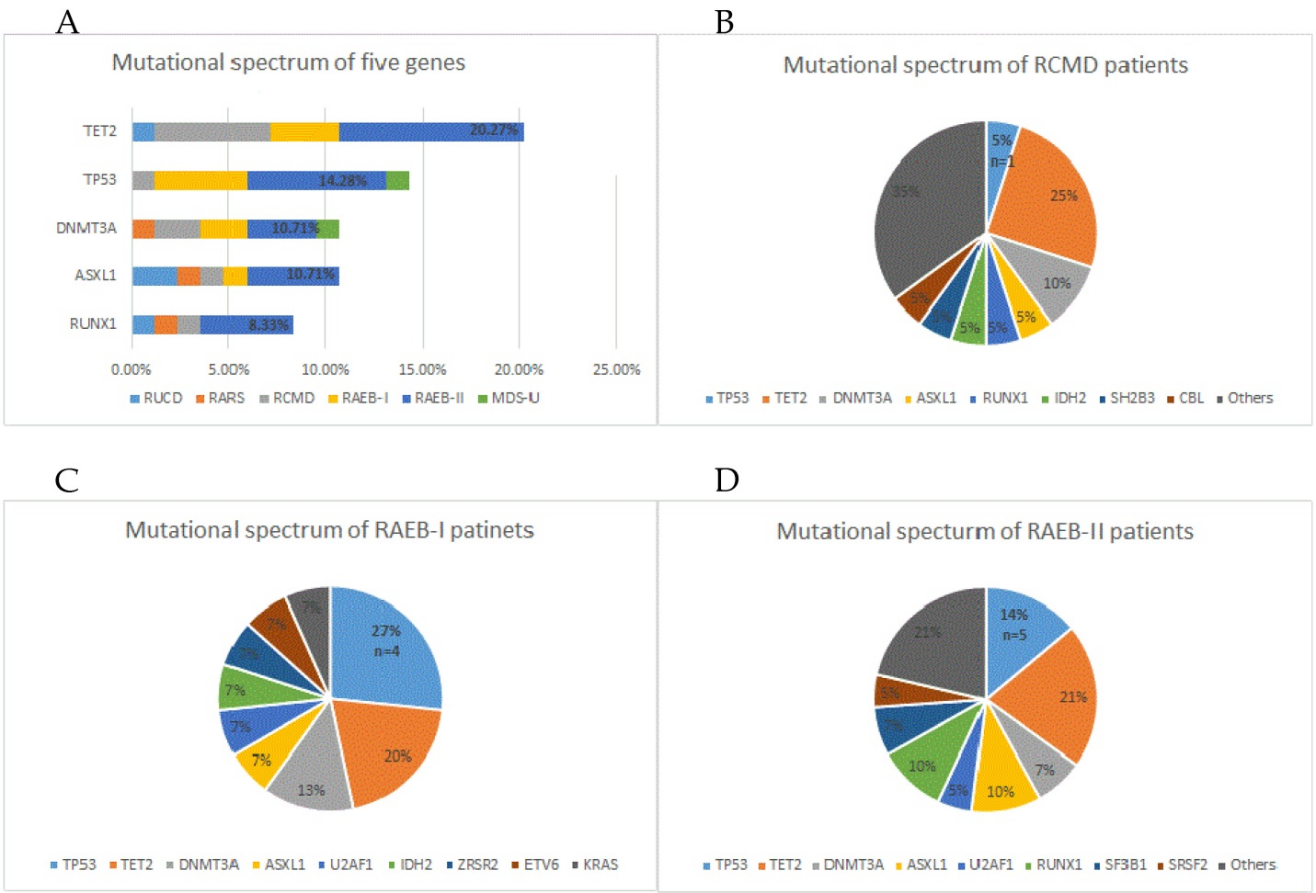
Gene mutation status in 84 study cohort patients that received next generation sequencing test. (A) Mutation status of five prime genes in 84 gene sequencing patients. (B) Mutation status of all MDS-related genes in RCMD patients. (C) Mutation status of all MDS-related genes in RAEB-I patients. (D) Mutation status of all MDS-related genes in RAEB-II patients.

To help improve the accuracy of risk stratification for MDS patients, 84 of all 110 study-cohort patients were subjected to gene sequencing. Fifty-three (63%) patients were found to have gene mutations, with 32 (29%) patients having merely one mutation, 15 (13.6%) having two mutations, 6 (5.5%) having no less than 3 mutations, and one patient having 9 gene mutations. The most frequently mutated genes were TET2, TP53, DNMT3A, ASXL1 and RUNX1 (Figure 2). We next analysis the distribution of the mutations and found that 82.8% were distributed in RCMD, RAEB-I and RAEB-II patients. The higher the risk of classification, the higher the quantity of TP53 mutations (Figure 3A). Moreover, TET2, TP53, DNMT3A and ASXL1 gene mutations occurred in high frequency in all three groups of patients (Figure 3B/C/D).

TET2, ASXL1 mutations were more likely to co-exist with other mutations, occurring at a ratio of 64.7% (χ2=20.516, P<0.0001) and 66.7% (χ2=19.329, P<0.0001), respectively. ASXL1 mutations tended to occur in patients with more mutations. In fact, 44.4% patients carrying ASXL1 mutation had 3 or more gene mutations (P<0.05). In contrast, with TP53 mutations the frequency of co-existence was 33.3% (χ2=12.726, P=0.013).

### Association between phenotypes and frequent mutations

Next, we investigated the association between phenotypes and frequent mutations. Indeed, correlations were found between mutations and phenotypes, such as age, HB/PLT/ANC level, karyotype, classification and IPSS/WPSS/IPSS-R risk level. The results showed that TP53 was associated with thrombocytopenia (r= -0.25, P=0.02), complex karyotype (r=0.38, P<0.001) and higher IPSS/IPSS-R risk level (r=0.35, P=0.001, r=0.33, P=0.002). TET2 mutation was associated with older age (r=0.23, P=0.035) and ASXL1 mutation with lower ANC level (r= -0.21, P=0.049).

### Associations between gene mutations and overall survival (OS)

**Figure 4:**
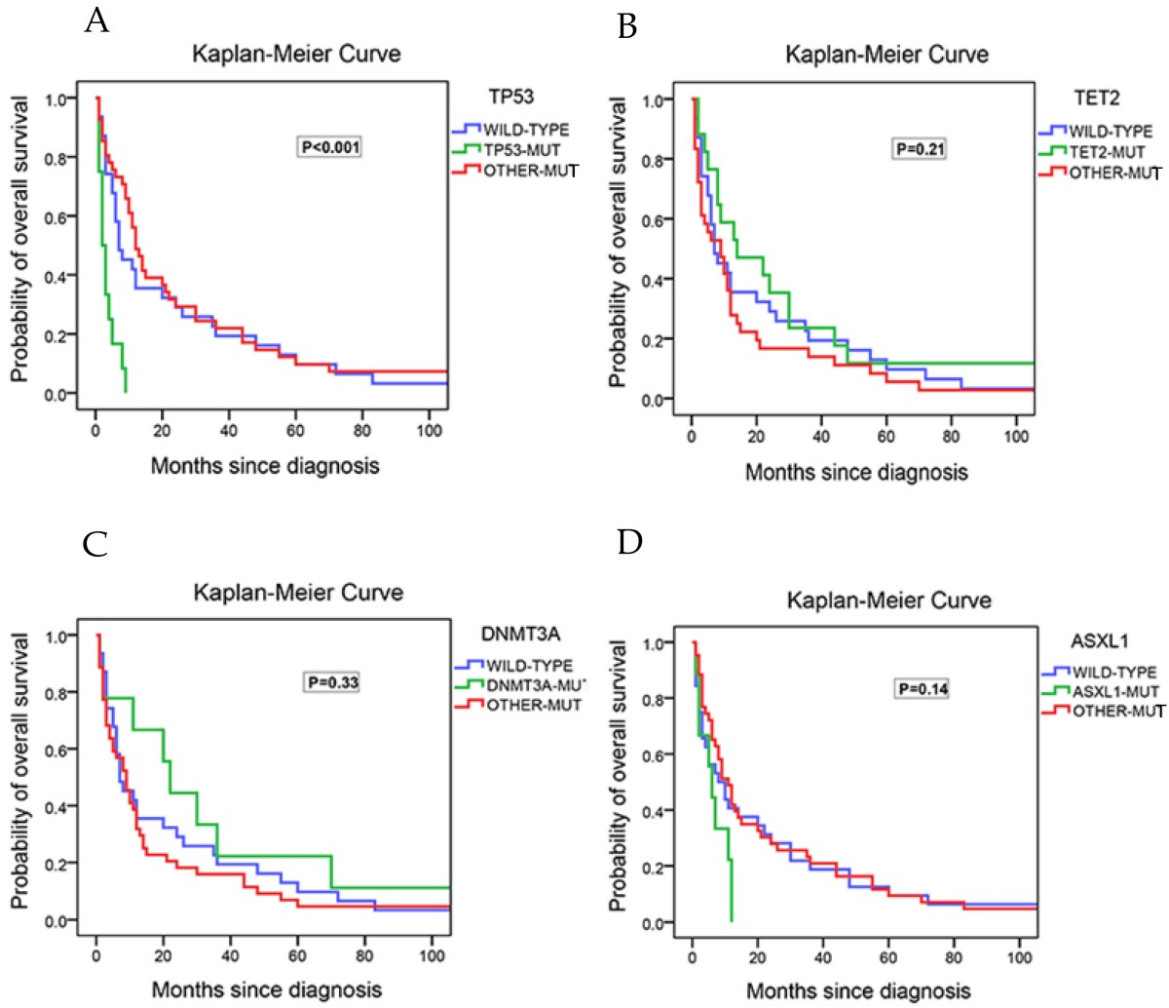
Kaplan-Meier curves for overall survival in the 84 study cohort patients that received next generation sequencing test. (A) Survival of patients with no mutation, with TP53 mutations and with the other mutations. (B) Survival of patients with no mutation, with TET2 mutations and with the other mutations. (C) Survival of patients with no mutation, with DNMT3A mutations and with the other mutations. (D) Survival of patients with no mutation, with ASXL1 mutations and with the other mutations.

The associations between gene mutations and OS was evaluated next. In univariate COX analysis, comparison between patients harboring TP53 mutations (14.3%) and those with wild-types (wt) showed that TP53 mutations were associated with a shorter overall survival (HR=5.67 [2.58-12.45], P<0.001; median OS, 2.5 months for patients with TP53 mutations vs. 11.5 months for those with wt). There was no statistical difference in the survival between patients who harbored mutated genes other than TP53 and the wild type patients (Figure 4A). Compared to wt patients, patients with TET2-mutation (20.3% of the patients; HR=0.68 [0.30-1.56], P=0.37), patients with DNMT3A-mutation (10.7% of the patients; HR=0.77 [0.27-2.17], P=0.62), or patients with ASXL1-mutation (10.7% of the patients; HR=0.54 [0.17-1.76], P=0.31) had no significant association with poor prognosis (Figure 4B/4C/4D). Taking both IPSS-R and TP53 mutation into multivariate analysis, the results showed that TP53 was significantly associated with overall survival (HR=5.25 [2.25- 12.25], P<0.001) regardless of the risk stratification. (Table 3) Addition of age to this model did not affect the overall results.

**Figure 5:**
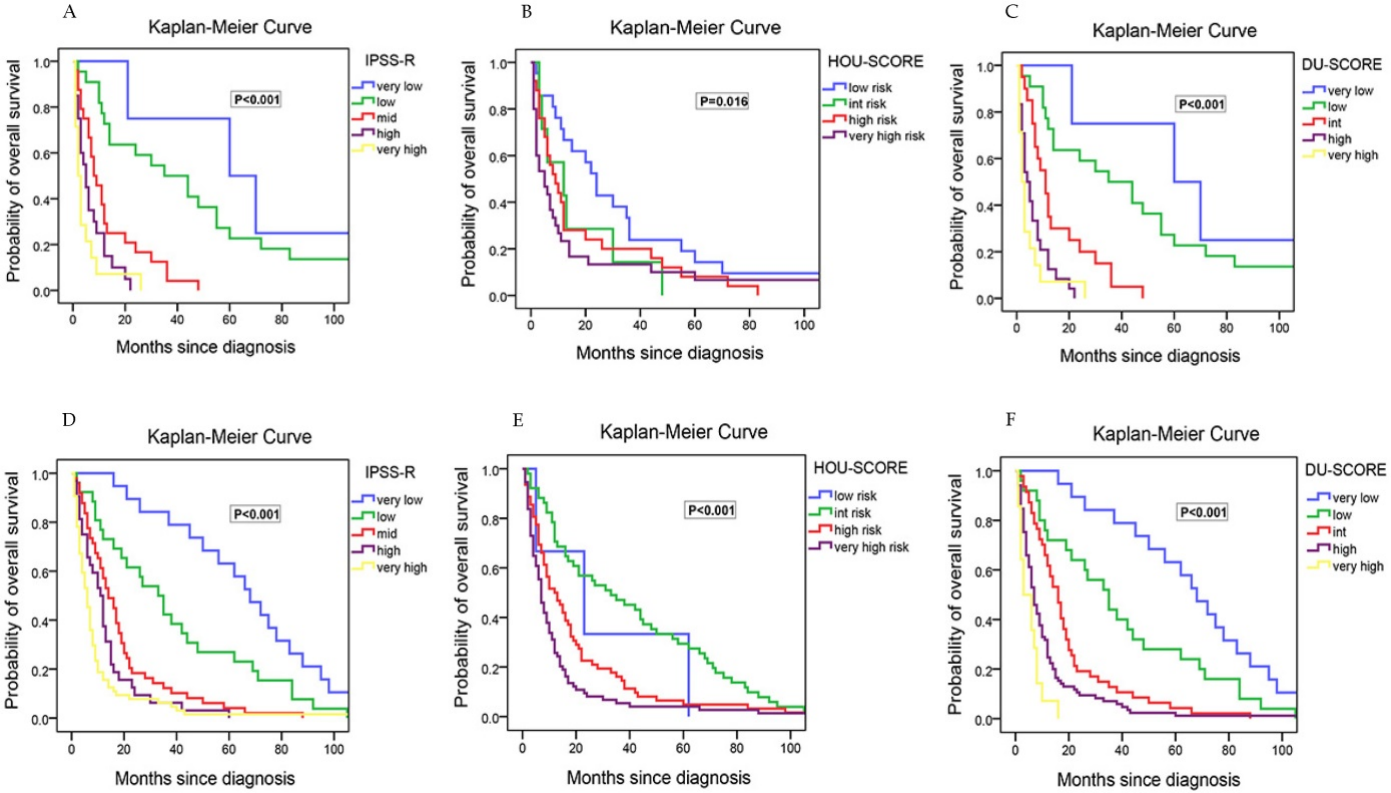
Kaplan-Meier curves for overall survival in MDS patients that received next generation sequencing test with survival data. Survival of 84 study-cohort patients: (A) according to IPSS-R. (B) according to HOU model. (C) according to DU model; Survival of 190 validation-cohort patients: (D) according to IPSS-R. (E) according to HOU model. (F) according to DU model.

In addition, a new risk model was evaluated in 84 NGS-patients in our study. Hou et al^17^ developed the risk model that incorporating the weighted coefficients of clinical and genetic factors (age ×0.025-IPSS-R lower risk group ×1.184 +CBL × 0.829+IDH2 ×0.829 +DNMT3A ×0.452 +ASXL1 × 0.442+TP53 ×2.254) for Chinese patients in 2018. Four risk groups were proposed: low (score <-0.5), intermediate (score -0.5~0.5), high (score 0.51~1.5) and very high (score >1.5). In our cohort, the median OS was 24, 12, 8 and 5 months for low, intermediate, high, and very high subgroups, respectively. The survival curve showed more intersections than IPSS-R (Figure 5A/B), and the mortality rate did not increase with risk level parallel.

To improve the risk stratification for MDS patients, an integrated scoring system based on our data incorporating IPSS-R and TP53 mutations into survival analysis was proposed. This model was developed incorporating the weighted coefficients of these two factors: TP53×1.658 + IPSS-R risk level×1.853. Five risk groups were proposed: very low (score < 2; n=4), low (score 2~5; n=21), intermediate (score 5.1~7; n=21), high (score 7.1~10; n =24), very high (score > 10; n=14). The median OS was 60, 35, 11, 4 and 2 months for very low, low, intermediate, high, and very high subgroups, respectively. This clinically relevant integrated scoring system divided the MDS patients into five groups with different clinical outcomes (P< 0.001) (Figure 5C). The median OS in relatively low-risk group of our model was longer than the same risk group of IPSS-R. Interestingly, patients who were in relatively low risk group according to IPSS-R but in relatively high-risk groups according to our new model were all TP53 mutated and cytogenetically normal.

To verify the prognostic value of IPSS-R, HOU model and our model, a validation cohort containing 190 next gene sequencing tested patients was then established. The validation cohort was made up by 104 males and 84 females with a median age of 52.9±15.5 years. Similarly, the patients concentrated on the classification of RCMD, RAEB-I and RAEB-II, and the overall mortality rate is also about 50%. Besides, the frequency of TP53/ TET2/ DNMT3A/ ASXL1/ RUNX1 gene mutation was high and the co-existing spectrum with TP53 mutation was narrow. Then, the three models (IPSS-R/ HOU model/ DU model) were assessed by these 190 patients accordingly. Our model was outperformed than IPSS-R and HOU model among OS, Kaplan-Meier curves and mortality rate. For IPSS-R, there was smaller discrepancy of OS between different risk stratified subgroups, and un-reasonable high mortality in high risk group (Table 2) than ours. For HOU model, a few patients were divided into low risk group (Table 2), which indicated that it could not figure out superior prognosis patients pretty well, let along more precise risk stratification of every subgroup for there was more crossover even than IPSS-R in Kaplan-Meier curves for overall survivals (Figure 5D/E/F).

## Discussion

This study showed that IPSS-R could better predict the prognosis in terms of mortality and overall survival of MDS patients, especially relatively low-risk MDS patients. Previous studies demonstrated that IPSS-R out-performed IPSS and WPSS in the prediction of transformation to acute leukemia and progression free survival (PFS) 20, 21. The better performance of IPSS-R might be attributed to finer stratification of Chromosomal karyotypes and quantification of peripheral blood loss. Initially, IPSS-R was used only for the establishment of primary patients before treatment, but not employed as a tool for therapy-related or prognosis monitoring of MDS^6^, ^22^, ^23^. However, studies found that IPSS-R worked well in treatment-related and post-treatment risk stratification, as well as dynamic prognosis monitoring in patients received lenalidomide or demethylating agents^23^-^26^.

Nevertheless, can IPSS-R alone be used in clinical practice? Indeed, IPSS-R still has its limitations. For example, WPSS has been proved to be effective in the prediction of OS, PFS, cumulative incidence of relapse (CIR) and cumulative incidence of non-relapse mortality (CINRM) in the post-transplantation patients 27. However, IPSS-R was only used in pre-transplantation assessment 28. In addition, for relatively low risk, so far researchers haven't agreed on whether IPSS-R or Low Risk Prognostic Scoring System (LR-PSS) 29 can better predict the prognosis. Previous studies showed that the two scoring systems were not satisfactory in identifying unfavourable OS, and LR-PSS was comparatively more sensitive than IPSS-R^8^, ^11^. In our study, the median OS of relatively low risk group was much shorter than the data provided by NCCN Guideline. Because patients with relatively low risk were given symptomatic and supportive treatment rather than chemotherapy or HMAs, the effect of treatment level on outcome should be ruled out. Therefore, there might be patients with poor prognosis mixed into relatively low risk groups.

With the development of gene sequencing technologies, more and more gene mutations were identified to be associated with MDS. Several studies revealed that the mutation spectra of MDS and some of the mutations were intimately related to the prognosis of MDS 30, 31. In this study, TP53 gene mutation was found to be associated with thrombocytopenia, complex karyotypes and reduced OS. It has been generally accepted that TP53 mutation is an independent factor for poor prognosis^32^-^34^ in MDS patients. Moreover, we assessed the prognostic impact of TP53 mutations on IPSS-R in MDS patients. So far there have been few studies integrating molecular data into IPSS-R in MDS patients^17^, ^30^, ^33^. Haferlach et al.^30^ utilized a combination of conventional factors (age, gender, and IPSS-R) and mutations in 14 genes (including TP53) as a novel prognostic model which had not been published in detail. The patients were stratified into four risk groups according to this model, which was demonstrated to be more accurate than IPSS-R in validation cohort. In a study of Nazha et al.^33^, incorporation of three mutations (EZH2, SF3B1, and TP53) into IPSS-R could improve the predictive power in 508 USA patients with primary and secondary MDS. Recently, Hou et al.^17^ developed an integrated risk-stratification model incorporating conventional risk factors (age and IPSS-R) and 5 gene mutations (CBL, IDH2, DNMT3A, ASXL1, TP53), which might be suitable for Chinese patients. Actually, the implication of mutations on clinical outcomes in Asian MDS patients may be different from that in western patients, due to difference in disease natures of MDS and racial back-ground between these two populations^13^-^15^. We next evaluated if the model established by HOU (HOU model) was suitable for MDS patients in our cohort. However, the results showed that patients could be better stratified by IPSS-R than the HOU model in aspect of survival analysis, median OS and mortality rate. Therefore, to improve prognostic prediction in Chinese MDS patients, we here proposed a new prognostic model incorporating TP53 mutations and IPSS-R based on our data. Our model can distinguish poor outcome patients with normal karyotype from real low-risk patients, thus indicating better prognostic power than IPSS-R, which have been verified in both study and validation group.

Apart from TP53, impact of most MDS-related gene mutations on the MDS prognosis remain controversial. One important reason is ethnic variation. For example, ASXL1 mutation did not predict outcome but only a trend of adverse prognosis of patients with MDS in our cohort and other Chinese population, in contrast, ASXL1 mutation was related to poor prognosis in European patients 12-15. Among the mutated genes, TET2, SRSF2 and SETBP1 have been subjected to META analysis 35-37 for no unanimous effect of them on prognosis reached. Moreover, co-mutations of genes further complicate the analysis and the interpretation of their influence on MDS prognosis. Our present study showed that TET2, ASXL1 and DNMT3A mutations were common in MDS patients (with mutation ratios 20.3%, 10.7% and 10.7%, respectively) and they all had co-mutations, but they were not found to be correlated with MDS prognosis. Patients with TET2 or DNMT3A mutations showed a higher OS rate at the beginning of the disease course when compared with other groups, probably because a large part of our patients received decitabine as main therapy. Of note, some studies reported that patients with TET2 and DNMT3A mutations had higher rates of response to HMAs and those with ASXL1 mutation had longer OS 38-40. Nonetheless, the impact of these mutations on the prognosis was moderate. Our further study will aim at identifying mutations that affect the efficacy of HMAs and prognosis of MDS and understanding the impacts they exert on the efficacy and prognosis.

The scoring systems of prognosis are designed to provide guides or information for better treatment. MDS patients with TET2 mutation could predict better response and a trend for longer OS to hypomethylating agents 38. However, the OS showed completely no difference between those with and without TET2 mutation when treatment was not taken into consideration^35^. Therefore, the effect of gene mutation is varied due to non-uniform therapeutic strategies. Until now, most meta-analyses didn't take into consideration the impact of treatment protocols but only analysed the general effect of gene mutations on prognosis, which is an important reason why a consensus has not been reached. Future studies should, on the basis of finer stratification, focus on the relationship between gene mutations (and other factors) and different treatment protocols.

The limitation of our study is that the total number of patients were not that variety, and all patients involved in were from one center but not from multicenter. Our ongoing work also aims to explore the outcome of gene mutation on outcome when treated with specific treatment.

In summary, IPSS-R is a scoring system that well fits Chinese MDS patients. A population-specific scoring system that integrates the second-generation gene sequencing technologies can help achieve more accurate prognostic assessment and thereby provide guidance for more effective treatment for Chinese MDS patients.

## Figures and Tables

**Table 1:** Clinical characteristics of the 110 Chinese MDS patients in the study cohort, 84 patients that received next generation sequencing test, the groups of patients divided by TP53 mutation status, and the 190-patients validation cohort.

	Study cohort	NGS ^a^ patient in study cohort	TP53-MUT in study cohort	TP53-WILD in study cohort	P value ^e^	Validation cohort
**Patients**	110	84	12	72	-	190
**Age(year)**	49.5±15.8	50.2±1.85	51.5±4.48	50.0±2.03	0.78	52.9±15.5
**HB(g/l)**	69.5(58.0-92.8)	93.2(67.5-110.5)	62.5(51.0-73.0)	68.5(59.0-96.8)	0.08	76.4(63.0-112.1)
PLT(*10^^9^/l)	45.5(21.0-121.1)	50.5(21.0-138.5)	32.5(18.5-63.3)	58(21.0-149.3)	0.08	49.2(38.1-130.2)
ANC(*10^^9^/l)	1.12(0.63-2.13)	1.20(0.64-2.43)	0.73(0.27-1.28)	1.44(0.72-2.51)	**0.02**	1.45(0.78-2.77)
**Classification ^b^**					0.58	
**RCUD ^c^**	9	7	0	7		13
**RARS** **RCMD**	2 26	2 17	01	2 16		8 43
**RAEB-I**	28	24	4	20		49
**RAEB-II**	38	30	6	24		66
**MDS-U**	7	4	1	3		9
**5q-**	0	0	0	0		2
**All**	110	84	12	72		190
**Karyotype ^d^**					**0.001**	
**Normal**	71	53	3	50		83
**1 abnormal**	23	16	2	14		62
**2 abnormal**	3	2	1	1		11
**≥3 abnormal**	13	13	6	7		34
**IPSS**					**0.001**	
**Low**	6	5	0	5		31
**Intermediate-1**	49	35	2	33		50
**Intermediate-2**	46	36	5	31		40
**High**	9	8	5	3		69
**WPSS**					0.23	
**Very Low**	2	1	0	1		17
**Low**	8	7	0	7		17
**Intermediate**	27	19	1	18		39
**High**	52	42	7	35		63
**Very High**	14	12	4	8		45
**MDS-U**	7	3	0	3		9
**IPSS-R**					**0.002**	
**Very Low**	4	4	0	4		19
**Low**	32	22	0	22		26
**Intermediate**	34	24	4	20		49
**High**	26	20	2	18		32
**Very High**	14	14	6	8		64
**OS**	11.0(4.0-30.0)	9.0(3.0-25.5)	2.5(1.3-4.8)	11.5(5.0-33.8)	**<0.001**	12.0(5.0-27.0)
**State**					**0.01**	
**live**	59	46	2	44		112
**die**	43	31	9	22		69
**sAML**	8	7	1	6		9

**RCUD**: refractory cytopenia with unilineage dysplasia; **RARS**: refractory anemia with ring sideroblast; **RCMD**: refractory cytopenia with multilineage dysplasia; **RAEB-I**: refractory anemia with excess blasts-I; **RAEB-II**: refractory anemia with excess blasts-II; **MDS-u**: myelodysplastic syndromes, unclassifiable; **5q**-: MDS associated with isolated del (5q); **sAML**: secondary acute myelocytic leukemia; **PB**: peripheral blood; **HB**: hemoglobin; **PLT**: platelet count; **ANC**: absolute neutrophil count **a**: 84 patients who were given target next gene sequencing (NGS) out of all 110 study cohort patients **b:** all 110 Chinese MDS patients were classified by WHO (2008) criteria **c**: RCUD includes refractory anemia (RA); refractory neutropenia (RN); refractory thrombocytopenia (RT) **d**: the risk group of cytogenetics in IPSS and IPSS-R are different. For IPSS, Cytogenetics: Good = normal, -Y alone, del(5q) alone, del(20q) alone; Poor = complex (≥3 abnormalities) or chromosome 7 anomalies; Intermediate = other abnormalities. For IPSS-R, Cytogenetic risks: Very good = -Y, del(11q); Good = normal, del(5q), del(12p), del(20q), double including del(5q); Intermediate = del(7q), +8, +19, i(17q), any other single or double independent clones; Poor = -7, inv(3)/t(3q)/del(3q), double including -7/del(7q), complex: (3 abnormalities); Very poor = complex: >3 abnormalities. Before the prognostic scoring system, which could fit Chinese better, were identified, we divided our patients only by number of cytogenetic abnormalities, other than any of IPSS or IPSS-R. **e**: Proportions of TP53-mut and TP53-wild patients were compared by utilizing Chi-square test. A P<0.05 was considered to be statistically significant.

**Table 2:** The mortality and median overall survival of different groups stratified according to IPSS/ WPSS/ IPSS-R/HOU model/DU model respectively

THE OUTCOME OF DIFFERENT PROGNOSTIC SCORING SYSTEM									
SYSTEM	NO.	RATE	FATAL	MORTALI TY	MEDIAN OS(m)	95% CI		Tarone-Ware	*P*
**IPSS (STUDY COHORT)**						LL	UL		
**low**	6	0.05	2	0.33	55	15.0	95.0	30.847	<0.001
**int-1**	49	0.45	12	0.24	20	11.8	28.2		
**int-2**	46	0.42	22	0.48	6	3.2	8.9		
**high**	9	0.08	7	0.78	2	0	4.9		
**all**	110		43						
**WPSS (STUDU COHORT)**									
**very low**	2	0.02	1	0.50	34	0.0	85.4	31.584	<0.001
**low**	8	0.07	1	0.13	33	25.9	156.1		
**int**	27	0.25	8	0.30	7	21.4	49.3		
**high**	52	0.47	23	0.44	3	9.5	21.0		
**very high**	14	0.13	9	0.64	2	1.9	8.8		
***MDS-U ^a^***	7	0.06	1	0.14	20	0.0	50.8		
**all**	110		43						
**IPSS-R (STUDY COHORT)**									
**very low**	4	0.04	1	0.25	60	12.0	108.0	55.854	<0.001
**low**	32	0.29	9	0.28	30	9.2	50.8		
**int**	34	0.31	13	0.38	11	6.7	15.3		
**high**	26	0.24	12	0.46	5	2.5	7.5		
**very high**	14	0.13	8	0.57	2	0.8	3.2		
**all**	110		43						
**IPSS-R (84 NGS of 110 STUDY COHORT)**									
**very low**	4	0.05	0	0.00	60	12.0	108.0	47.489	<0.001
**low**	22	0.26	2	0.09	35	12.0	58.0		
**int**	24	0.29	8	0.33	8	4.2	11.8		
**high**	20	0.24	11	0.55	5	2.8	7.2		
**very high**	14	0.17	10	0.71	2	0.8	3.2		
**all**	84		31						
**HOU Model (84 NGS of 110 STUDY COHORT)**									
**low**	21	0.25	5	0.24	24	18.1	30.0	10.338	0.016
**int**	7	0.08	3	0.43	12	0	27.4		
**high**	26	0.31	10	0.38	8	3.0	13.0		
**very high**	30	0.36	13	0.43	5	0.7	9.3		
**all**	84		31						
**DU Model (84 NGS of 110 STUDY COHORT)**									
**very low**	4	0.05	0	0.00	60	12.0	108.0	52.256	<0.001
**low**	22	0.26	2	0.09	35	12.0	58.0		
**int**	20	0.24	6	0.30	11	6.6	15.3		
**high**	24	0.29	13	0.54	4	1.9	6.1		
**very high**	14	0.17	10	0.71	2	0.8	3.2		
**all**	84		31						
**IPSS-R (VALIDATION COHORT)**									
**very low**	19	0.10	3	0.16	68	53.8	82.2	81.437	<0.001
**low**	26	0.14	6	0.23	33	21.8	44.2		
**int**	49	0.26	16	0.33	14	9.4	18.6		
**high**	32	0.17	19	0.59	11	7.8	14.2		
**very high**	64	0.34	34	0.53	6	4.4	7.6		
**all**	190		78						
**HOU Model (VALIDATION COHORT)**									
**low**	3	0.00	1	0.33	23	0.0	51.8	39.864	<0.001
**int**	51	0.27	14	0.27	33	12.0	54.0		
**high**	62	0.33	25	0.40	12	6.9	17.1		
**very high**	74	0.39	38	0.51	7	5.3	8.7		
**all**	190		78						
**DU Model (VALIDATION COHORT)**									
**very low**	19	0.10	3	0.16	68	53.8	82.2	87.109	<0.001
**low**	25	0.13	6	0.24	35	22.0	48.0		
**int**	47	0.25	15	0.32	16	12.6	19.4		
**high**	85	0.45	44	0.52	7	5.3	8.7		
**very high**	14	0.07	10	0.71	3	0.0	6.7		
**all**	190		78						

**IPSS:** International Prognostic Scoring System; **WPSS**: WHO Prognostic Scoring System; **IPSS-R**: Revised International Prognostic Scoring System; **NGS patients**: patients that received next generation sequencing test; **HOU model**: the prognostic model for MDS established by HOU and his colleague; **DU model**: the prognostic model for MDS established based on our cohort; **LL:** low-limit; **UL:** up-limit **a:** the classification of *MDS-U* can't be stratified by WPSS

**Table 3:** Univariate and multivariate analysis (Cox regression) for the overall survival in 84 study-cohort MDS patients that received next generation sequencing test

**Univariate COX analysis**		**P**		**HR**	**95 CI**
**TP53**		**<0.001**		5.67	2.58-12.45
**TET2**		0.37		0.68	0.30-1.56
**DNMT3A**		0.62		0.77	0.27-2.17
**ASXL1**		0.31		0.54	0.17-1.76
**RUNX1**		0.21		0.28	0.04-2.02
**IPSS-R**		**<0.001**		7.11	2.42-20.91
				
**Multivariate COX analysis**		**P**	**B**	**HR**	**95 CI**
**All patients**	TP53	<0.001	1.658	5.25	2.25- 12.25
	IPSS-R	0.001	1.853	6.38	2.15- 18.93
**Relatively low-risk patients**	TP53	0.020	1.656	5.24	1.30-21.09
	IPSS-R	<0.001	1.658	5.25	2.06- 13.38
**Relatively high-risk patients**	TP53	0.001	1.22	3.38	1.62- 7.07
	IPSS-R	0.003	1.20	3.32	1.49- 7.38
